# Use of a Tablet Attachment in Teleophthalmology for Real-Time Video Transmission from Rural Vision Centers in a Three-Tier Eye Care Network in India: eyeSmart Cyclops

**DOI:** 10.1155/2019/5683085

**Published:** 2019-04-01

**Authors:** Abhinav Loomba, Sandeep Vempati, NavyaDeepthi Davara, M. Shravani, Priyanka Kammari, Mukesh Taneja, Anthony Vipin Das

**Affiliations:** ^1^Department of Teleophthalmology, L V Prasad Eye Institute, Hyderabad, India; ^2^Department of Cornea & Anterior Segment, L V Prasad Eye Institute, Hyderabad, India; ^3^LVPEI Center for Innovation, L V Prasad Eye Institute, Hyderabad, India; ^4^Department of eyeSmart EMR & AEye, L V Prasad Eye Institute, Hyderabad, India

## Abstract

**Purpose:**

This study describes the development of an attachment for a smart tablet to stream live video information of an eye examination through the slit lamp.

**Methods:**

A tablet attachment was developed that enables the live streaming of video from the slit lamp from the rural vision centers of the LVPEI network. A video streaming solution like Skype Lite was used to transmit the same. The eyeSmart app was utilized for the documentation of the clinical information of the patients.

**Results:**

A tablet attachment of eyeSmart Cyclops was developed and piloted in 3 vision centers of the LVPEI network.

**Conclusion:**

The use of real-time video transmission illustrates a novel teleophthalmology solution in low resource settings to screen rural populations. The ability to transmit live video enables gathering more information than static images.

## 1. Introduction

Teleophthalmology combines technology and medicine and can help facilitate the delivery of eye care to rural areas. In the field of ophthalmology, a slit lamp biomicroscope is used as a fundamental diagnostic device. For wider outreach, teleophthalmology is gaining interest as it saves the patient money and time to travel to seek the opinion of an ophthalmologist [1–5]. Smartphones and tablets are used currently to gather images of the patient's eye and these are sent to a consulting doctor for further diagnosis and plan of management [6].

The practice of clicking and sending images or videos using a smartphone is used by many in the field of teleophthalmology; however, use of a tablet is limited and the use of real-time video transmission is evolving [6].

This paper presents a novel method of the use of a tablet attachment called “eyeSmart Cyclops” for real-time video transmission from rural vision centers in an eye care network in India.

## 2. Materials and Methods

The eyeSmart EMR app was launched in 2016 to digitize the rural vision centers and the app has enabled over 501,771 consultations over the past 24 months. Teleophthalmology and video calling are an added service through the EMR. LV Prasad Eye Institute (LVPEI), a comprehensive eye health facility with its Centre of Excellence located in Hyderabad, is a nongovernment eye care institution. It has 3 tertiary care centers, 18 secondary centers, and 176 vision centers. One vision center serves a population of 50,000 and is linked to the secondary centers which in turn are linked to the tertiary care centers.

Every vision center has a trained vision technician who has undergone training at LVPEI, Hyderabad, for a minimum period of 1 year. Every vision center is equipped with a slit lamp biomicroscope (Carl Zeiss SL 115). Every vision center through a tablet (iBall Slide Brace XJ) is connected online through the cloud by the eyeSmart EMR app. The tablet runs on a 3G network (Idea 3G prepaid). The registration details and clinical data of patients who are visiting the vision centers are entered into the EMR app. Slit lamp photographs of the patients requiring a teleophthalmology consult are captured using the tablet. The photographs are then attached through the EMR app and synced online for a consult through the cloud. The Teleophthalmology Command Center situated at the Centre of Excellence in Hyderabad receives the request in real time and has access to the EMR app of all vision centers across the network.

“eyeSmart Cyclops” is an in-house developed attachment for the iBall Slide tablet.

## 3. Design Process of eyeSmart Cyclops

**Figure 1:**
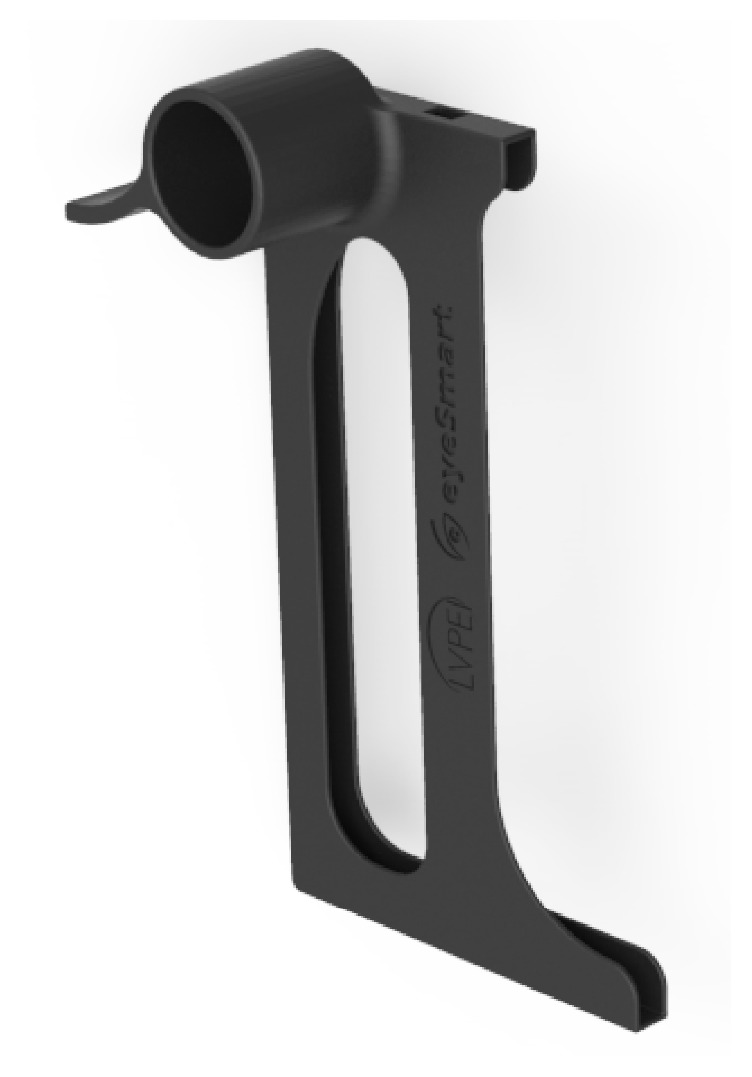
CAD design of eyeSmart Cyclops.

The things that should be taken into consideration while designing Cyclops are as follows (Figure 1):

1. It should have a good hold while attached on the slit lamp
2. The imaging should be done in such a way that the camera covers the maximum field of view of the eye
3. As the user frequently needs to mount and unmount the case from the slit lamp, it should be completely cased as there is lot of device movement at the vision center
4. The case should be sturdy to withstand wear and tear in the clinic

### 3.1. Grip (Figure 2)

The eyepiece holder is extruded to sufficient length of 45 mm to grip the eyepiece of the slit lamp properly and secure it while working and moving the slit lamp. The inner lining of Cyclops snaps on to the eyepiece to a length of 30 mm giving it the strength for attachment.

**Figure 3:**
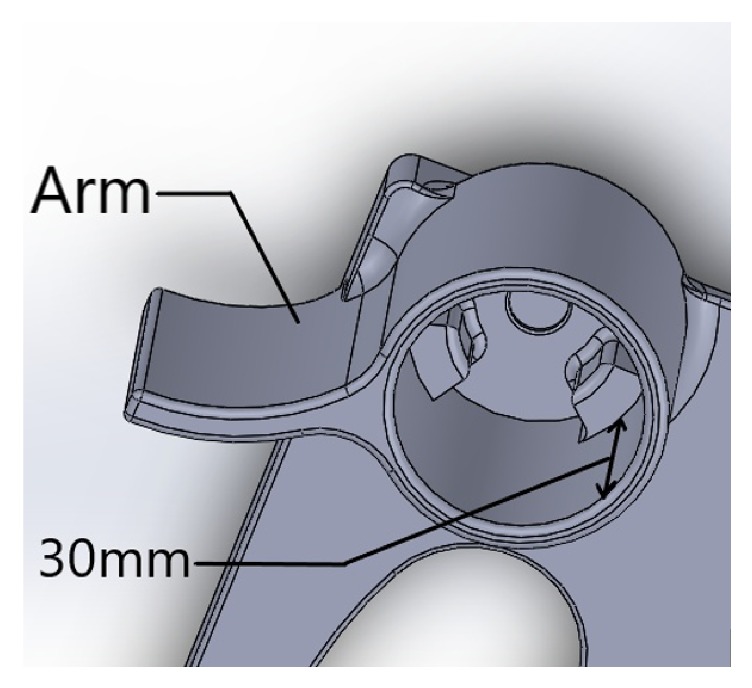
Side arm of Cyclops.

The whole device gets its rigidity from the side arm (Figure 3) which rests underneath the fellow eyepiece. The arm helps the case to hold it in place with the need of fixing it to the slit lamp unlike existing mobile mounts. This also eases the egress of the case from the slit lamp.

### 3.2. Imaging

**Figure 4:**
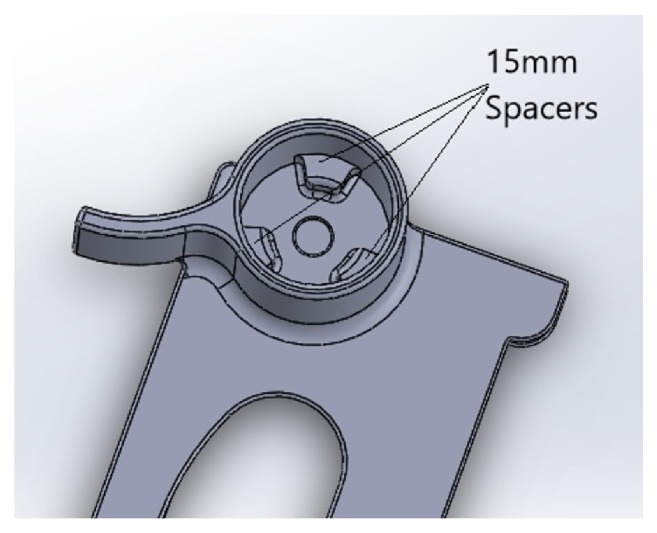
Spacers in the eye grip.

To image the findings on the slit lamp at maximum resolution, the camera of the iBall tablet needs to cover the maximum field of view through the objective 20x magnification eyepiece. To achieve this it was found that the camera has to be placed 15 mm from the eyepiece. So 3 spacers are provided inside the extruded eye grip to space it 15 mm away from objective lens (Figure 4).

### 3.3. Sliding Mechanism (Figure 5)

With the whole case being protruded at the camera it is wise to remove it while it is not in use, which would in turn increase the life period of the case. For this a sliding mechanism is provided in the case so that when required the tablet can slide into the case and then it can be mounted on the eyepiece.

### 3.4. Wear and Tear

**Figure 6:**
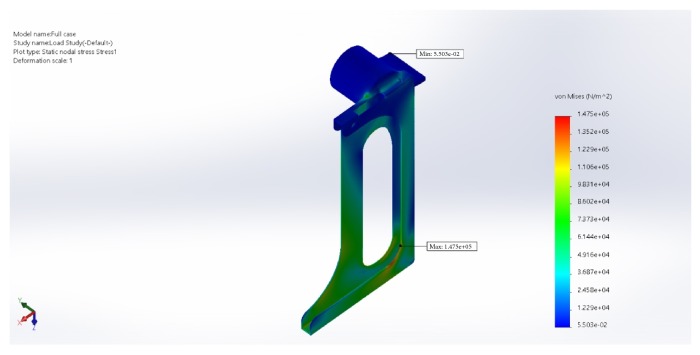
Static stress plot (the force is transmitted equally towards all features of the design).

**Figure 7:**
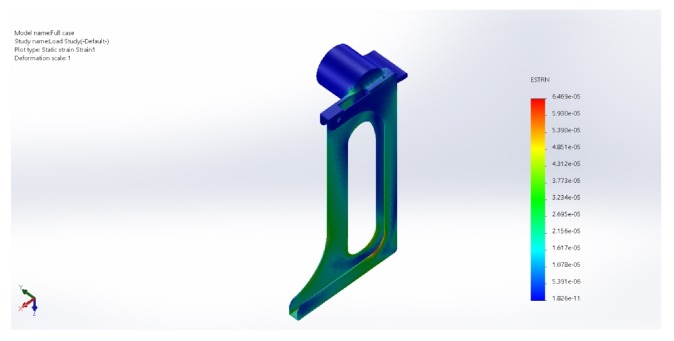
Static strain plot (as the stress is equally distributed we can see no built up strain across the design).

**Figure 8:**
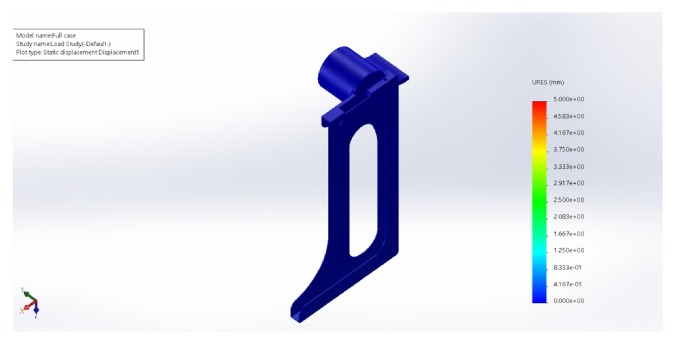
Static displacement plot (we can see that for a load of 200 gms the maximum displacement is no more than 0.02 mm).

Simulations have been made to designs every time in the process and the features of the design were altered such that the case will be strong enough to hold the tablet (200 gms). The idea is to generate the least stress and displacement and good load distribution. By this constant upgradation, a complete stable design is finalized. Load distribution simulations have been depicted (Figures 6–8).

The chosen material properties are as follows:

  Name: ABS
  Model type: linear elastic isotropic
  Tensile strength: 3e+07 N/m∧2
  Elastic modulus: 2e+09 N/m∧2
  Poisson's ratio: 0.394
  Mass density: 1020 kg/m∧3
  Shear modulus: 3.189e+08 N/m∧2
  Load on the base = 200 gms

### 3.5. Fabrication

We used 3D printing to print the first 5 prototypes. This model was printed on a high end SLS 3D printer using the 3D printer's proprietary material nylon/PA12 (white opaque) and the tolerances were given accordingly. After getting printed these models were painted with matte finish paint which gives the whole rugged look.

**Figure 9:**
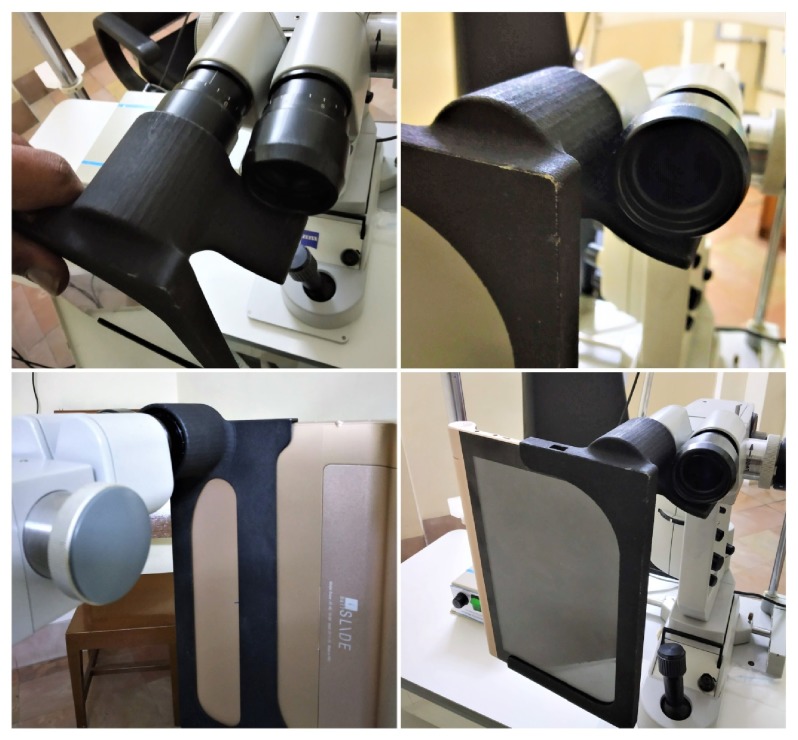
Cyclops attachment with the tablet on the slit lamp.

**Figure 10:**
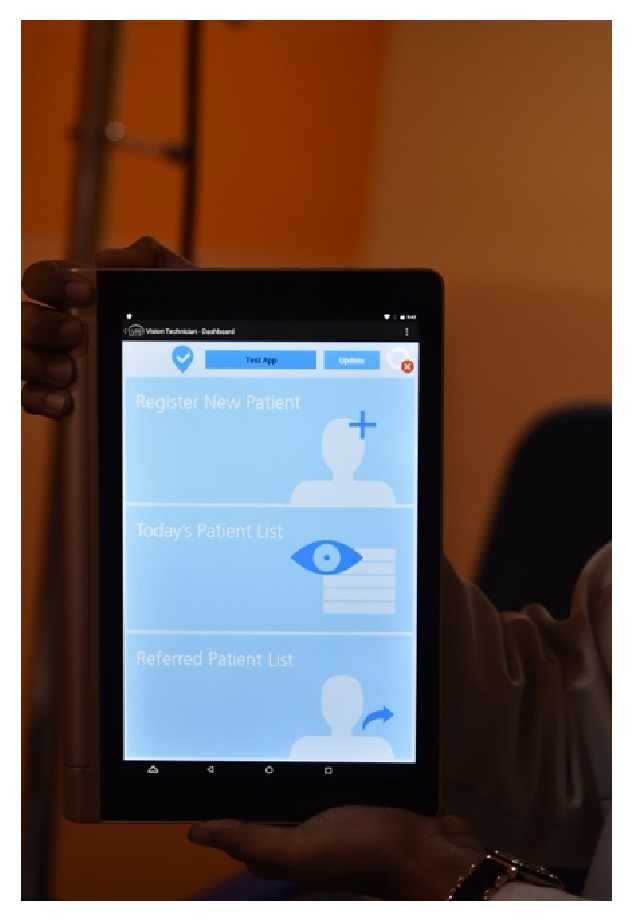
eyeSmart app on the tablet.

**Figure 11:**
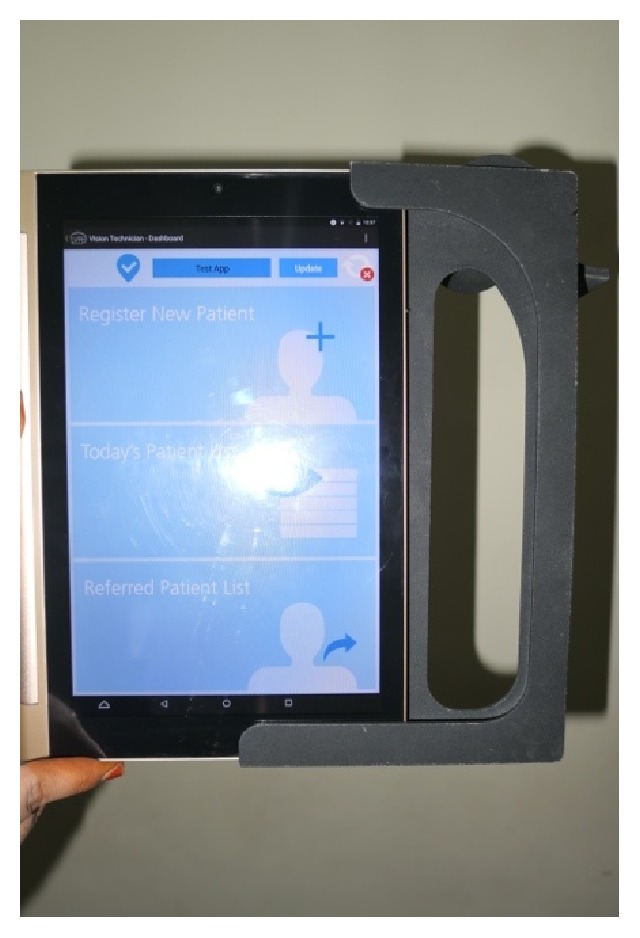
Insertion of tablet into eyeSmart Cyclops.

**Figure 12:**
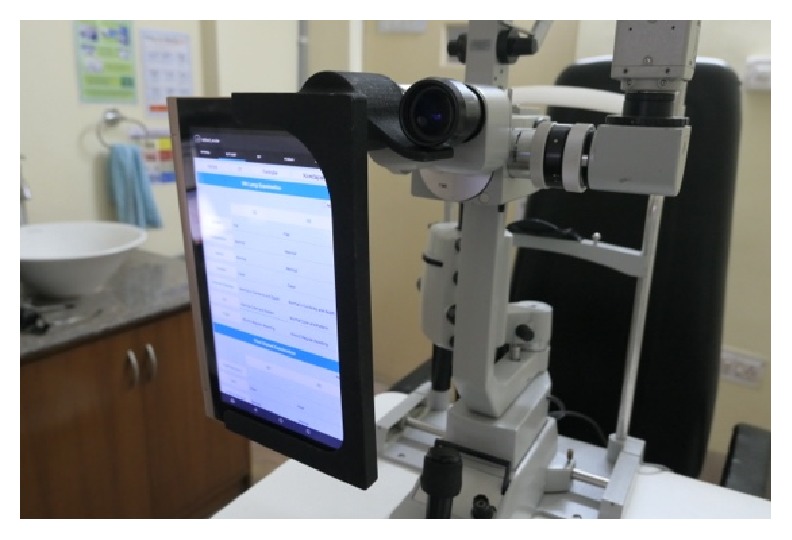
Placement of tablet with attachment.

**Figure 13:**
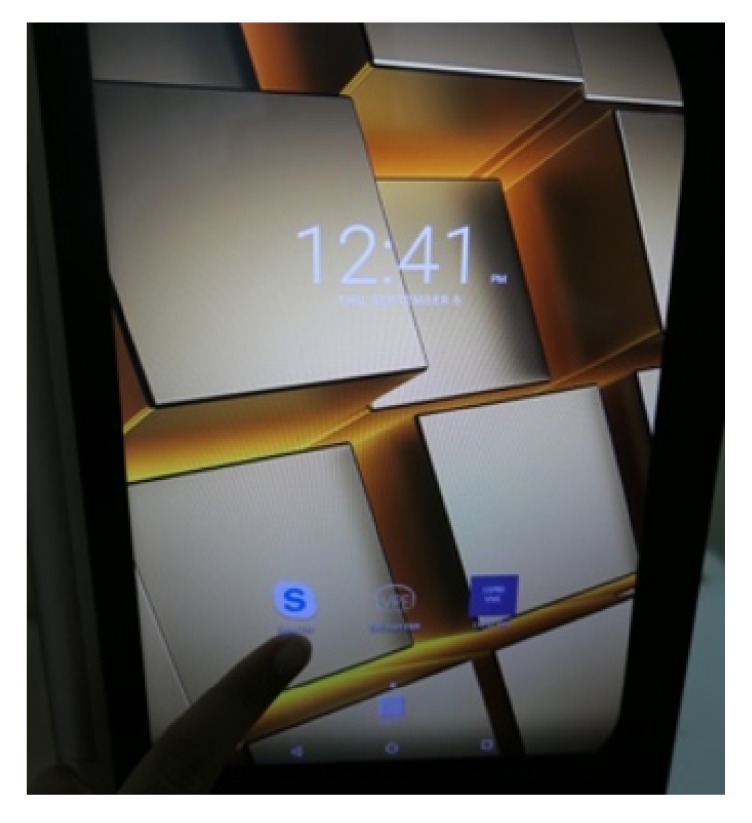
Use of Skype Lite on the tablet.

**Figure 14:**
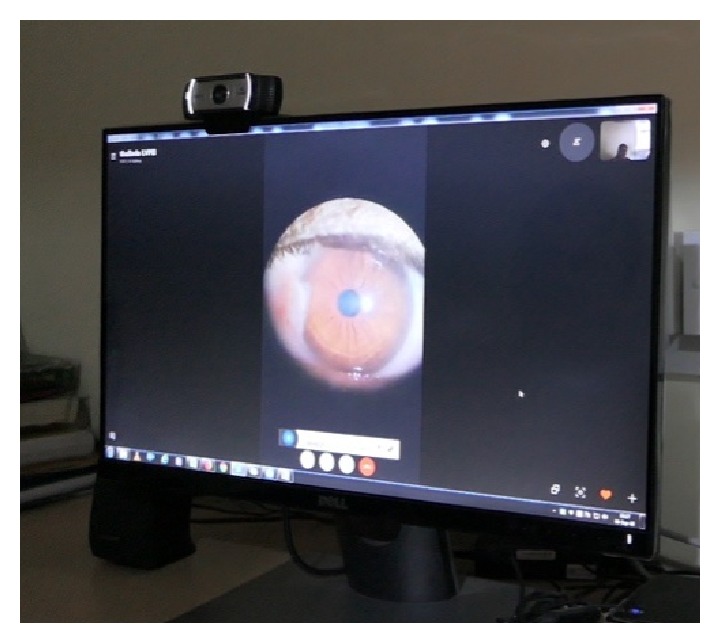
Command center receiving the call.

**Figure 15:**
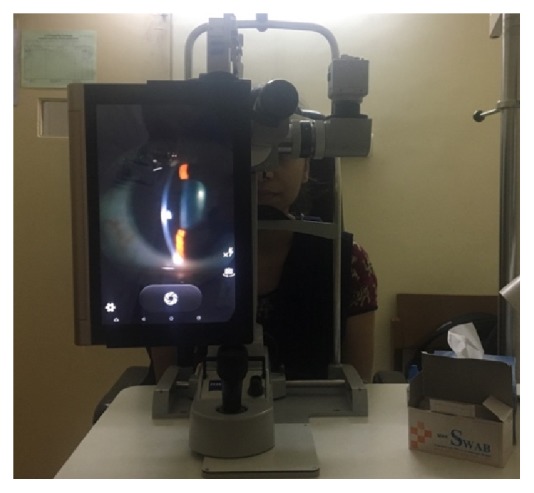
eyeSmart Cyclops attached to the slit lamp.

**Figure 16:**
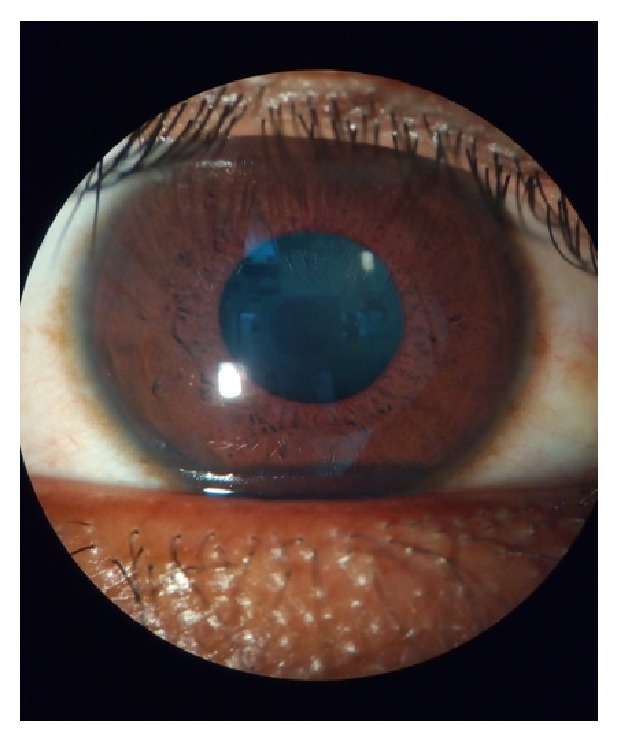
Magnified view of the eye through eyeSmart Cyclops.

**Figure 17:**
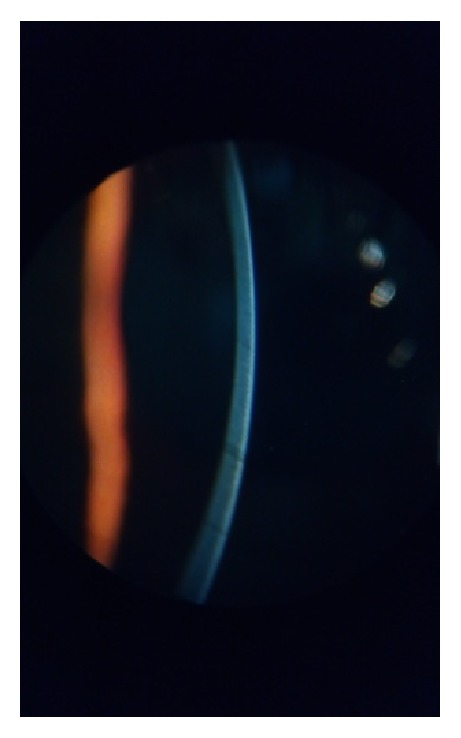
Slit view of the cornea through eyeSmart Cyclops.

“eyeSmart Cyclops” is attached to the eyepiece of the slit lamp (Figure 9). The patients are then connected via video call to the tertiary care center in Hyderabad using Skype Lite and the procedure was explained to the patient. The consultant doctor interacts with the patient regarding the history of his/her disease. After the call is placed the tablet is attached to the Cyclops attachment and the eye of the patient to be examined is focused while using real-time video transmission using the rear camera of the tablet. The vision technician is instructed regarding the focusing of a particular part of the eye in real time. Examination is carried out under diffuse illumination followed by slit examination of a particular area. With the help of live video transmission, examination of the patient's eye is performed. Photographs can be clicked when necessary. The consultant doctor interacts with the patient after the examination and recommends any medical or surgical intervention as required. The patient is also recommends referral to a secondary or tertiary center if required for further medical or surgical management (Figures 10–17).

**Figure 18:**
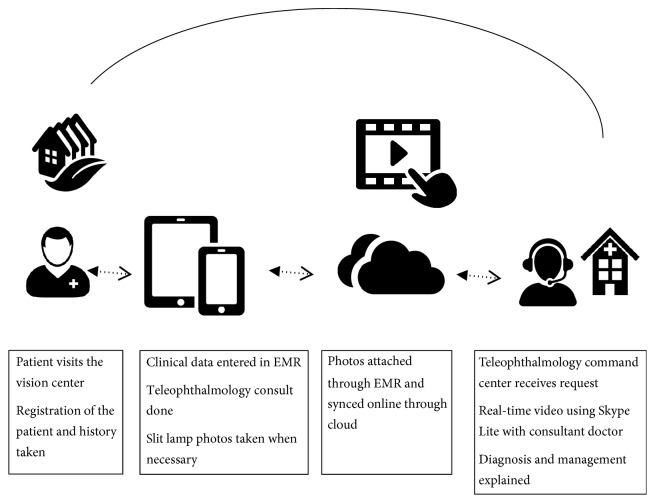
Steps in a vision center.

The various steps starting from registration of a patient in a vision center to a teleophthalmology consultation in the command center are described in Figure 18.

## 4. Discussion

The common approach in the field of teleophthalmology is to capture still or video images of the patient, where the images are acquired by a technician and sent to a different location for diagnosis and plan of management [7]. These images can be taken with the help of a smartphone or a tablet though a smartphone is the most commonly used device [8]. This reduces the referral rates and ultimately reduces costs for patients. Thus teleophthalmology has a promising impact on the reduction of inequalities in access to healthcare [1–5]. A remotely operated slit has been described which revolutionizes the use of a slit lamp in teleophthalmology; however, it is costly and needs experts to convert a conventional slit lamp into a slit lamp which can be remotely operated [9].

Teleophthalmology can be used for screening common ophthalmic diseases especially in developing countries where easy access to the experts is not available for all people, especially in rural areas [10].

The addition of Cyclops is a boon and real-time video transmission is the future of teleophthalmology. It provides excellent image quality where the consulting doctor can instruct the technician to focus on particular areas of interest which may be missed if only a photograph is sent. The whole eye can be examined in the process and treatment can be prescribed saving the patient a lot of money to travel to a tertiary care center. It will particularly be helpful for patients for whom regular follow-ups are required who lose daily wages to travel hundreds of kilometers for a consultation. It provides a very cheap alternative and all it requires is a good 3G/4G/Wi-Fi network, tablet, and a slit lamp. It overcomes the limitation of sending only a handful of pictures, allowing us to gather more information from the video as opposed to a 2-dimensional picture. It also frees up the hands of the vision technician to follow the instructions to alter the illumination and magnification required for gathering more information.

The only limitation to the proposed procedure is poor connectivity in the rural areas.

A real-time transmission of videos using a tablet has been described in literature with the use of a drone slit lamp [9]. However, it is expensive to modify the slit lamp and is limited by connectivity.

Our methodology can revolutionize teleophthalmology and reach even that strata of patients who would otherwise not be able to visit a consulting doctor.

## Figures and Tables

**Figure 2:**
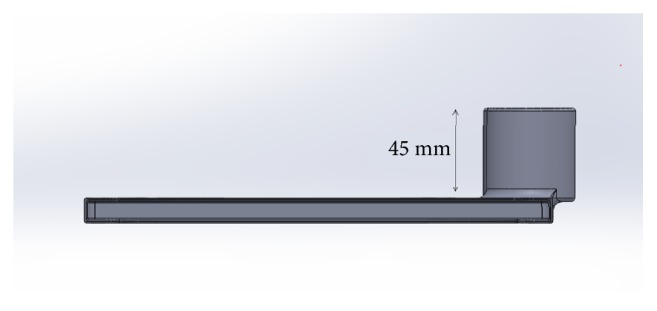
Grip of the Cyclops.

**Figure 5:**
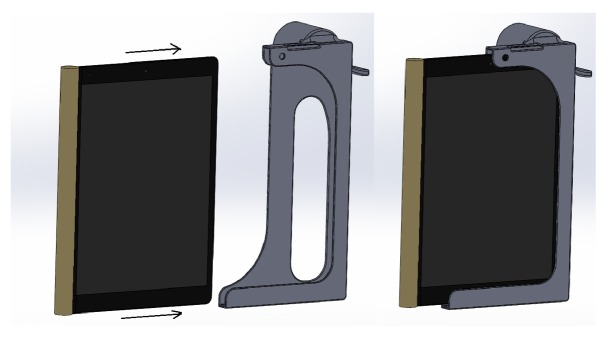
Sliding mechanism for the tablet.

## Data Availability

We did not include any statement related to data availability as this study describes the design and development of a tablet attachment in teleophthalmology for real-time video transmission from rural vision centers in a three-tier eye care network in India.
